# Patients’ experiences with the routine use of a clinical feedback system prior to consultations in ostomy care: a qualitative study

**DOI:** 10.1007/s11136-025-03916-z

**Published:** 2025-02-15

**Authors:** Lill Anette Juvik, John Roger Andersen, Kisten Lerum Indrebø, Anne Marie Sandvoll

**Affiliations:** 1 Department of Surgery, Førde Hospital Trust, Førde, Norway; 2 Department of Health and Caring Sciences, Western Norway University of Applied Sciences, Førde, Norway; 3 Department of Research and Innovation, Førde Hospital Trust, Førde, Norway

**Keywords:** Ostomy care, Clinical feedback system, Patient-reported outcomes, Patient-reported outcome measurements, Qualitative research

## Abstract

**Purpose:**

Ostomy surgery involves significant bodily changes, and the adjustment process encompasses a broad spectrum of physical and psychosocial challenges. A clinical feedback system (CFS) has been developed to collect patient-reported outcomes as part of routine outpatient follow-up, reviewed by stoma care nurses, to better address patients’ needs during their adjustment process. The intervention appears promising; however, empirical evidence supporting its benefits remains limited. Thus, we explored patients’ experiences with the routine use of the CFS prior to consultations in ostomy care.

**Methods:**

A qualitative design involved 27 semi-structured individual interviews with patients using CFS as part of routine care. The data were analysed using Braun and Clarke’s reflexive approach to thematic analysis.

**Results:**

The overarching theme *CFS—a tool with potential and multiple mechanisms of action* was developed with three themes: (1) Grasping the purpose can be challenging, (2) Preparatory learning—triggering reflection and self-awareness, and (3) Means of communication and potential for being understood. Engaging with the CFS had personal utility value with many dimensions, varying in strength and significance for each patient. Even though not everyone grasped the purpose of its use, it was part of a preparatory learning process for consultations and the adjustment process itself. It triggered reflection and self-awareness and served as a means of communication with potential for follow-up.

**Conclusion:**

Although the CFS appears promising, future research should focus on identifying the most effective methods for educating patients on its use.

**Supplementary Information:**

The online version contains supplementary material available at 10.1007/s11136-025-03916-z.

## Introduction

Approximately 3,700 ostomy surgeries are performed annually in Norway [1] due to conditions such as cancer, inflammatory bowel diseases (IBD), congenital abnormalities, and several other diagnoses [2]. An ostomy diverts urine or faeces to an external pouch, necessitating significant adjustments in bodily function and appearance [3, 4]. These changes can profoundly impact physical, psychological, and social aspects of life [5–9], as well as health-related quality of life (HRQoL) [4, 10].

There has been an increase in the routine collection and active use of patient-reported data in clinical care. To systematically measure patients’ progress, a clinical feedback system (CFS) with standardized self-reported items relevant to specific health conditions is often utilized. Patient-reported outcome measures (PROMs) are tools used to measure these patient-reported outcomes (PROs), and the results are made available for healthcare professionals to assess [11]. PRO data are integrated into clinical practice with a primary goal of fostering patient-centred care and treatment and allowing patients to participate actively in decisions about their health [12]. PROMs can help tailor care to individual needs, aid clinical decision-making, and inform value-based healthcare initiatives [13, 14].

Studies on the effectiveness of using PROs indicate moderate improvements in patient-clinician communication, with minor enhancements in QoL [15]. In mental healthcare, a small positive effect on treatment outcomes has been noted [16]. Furthermore, research in cancer care has predominantly shown positive outcomes, leading to better HRQoL, patient satisfaction, and patient-clinician communication [17]. Patients’ perspectives on using PROMs have been explored across various conditions and clinical settings. Systematic meta-syntheses have found that PROMs promote active patient involvement, enhance the focus of consultations, improve the quality of care, and strengthen patient-clinician relationships [18]. Additionally, PROMs facilitate self-reflection and effective communication [13], empowering patients and often fostering collaborative practice [19, 20].

Few intervention studies have addressed adjustment-related issues or provided insights into how follow-up can contribute to a new and altered normality [21]. A novel CFS with PROMs, called the Ostomy Adjustment System (OAS), has been developed for collecting patient-rated data as part of routine outpatient follow-up practice and is reviewed by stoma care nurses (SCNs) to better address patients’ needs in their adjustment process [22–24]. According to quantitative investigation of patients’ experiences with the CFS, patients are satisfied with their follow-up using pre-consultation questionnaires, receive sufficient and individualized information, are involved in treatment decisions, and benefit from the consultations [25]. Measured life areas such as daily activities, knowledge and skills, health, and the patient’s HRQoL improve during the first year after the operation. The CFS potentially improves overall well-being [25]. Thus, it is a promising method for follow-up, potentially promoting better discussions during consultations and tailoring the patient’s adjustment trajectory more precisely than without such a system.

However, to the best of our knowledge, no research has focused on patient experiences with such a CFS to aid the adaptation process in ostomy care. Consequently, there has been a call to evaluate it more thoroughly [24, 25]. Therefore, the aim of this study was to explore patients’ experiences with the routine use of the CFS prior to consultations in ostomy care.

## Methods and materials

### Context

The OAS was developed and standardized for the Norwegian population by Førde Hospital Trust [22]. It was initiated to allow patients and SCNs to prepare for consultations, thereby improving consultation quality, patient involvement, and adaptation to an ostomy. Items were generated based on patients and SCNs needs, then tested and refined in clinical development studies [22–24]. OAS was implemented at one outpatient clinic in 2017, and Version 2 was rolled out to three more hospitals within the same regional health authority in 2022. The system is administered electronically via smartphone, PC, or tablet [24].

**Fig. 1:**
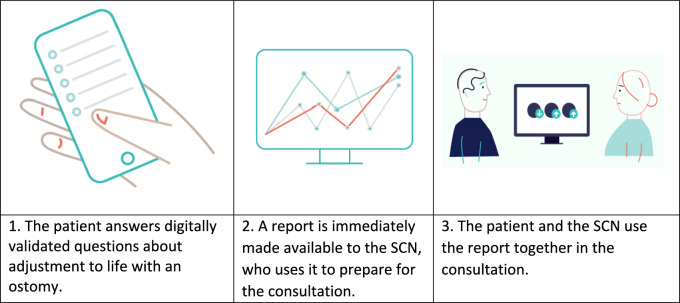
Use of the ostomy adjustment system

The follow-up according to the national recommendations should occur at 3 weeks, and at 3-, 6-, and 12-month postoperative intervals, and then annually, along with low-threshold services. Consultation includes 1) clinical control of the ostomy, skin and ostomy equipment, and aids, and 2) discussion with the patient, including health education and guidance [26]. Patients answer the CFS before each consultation, starting from 3 months onwards (Fig. 1.)

The CFS contains a sociodemographic and clinical form, the Ostomy Adjustment Scale [27] and the Coop-Wonca Chart [28]. Version 2 consists of up to 86 items distributed across multiple scales [23]. Patients respond on a six-point Likert scale for the Ostomy Adjustment Scale and a five-point scale for the Coop-Wonca Chart. The system generates visual reports with graphs and bars summarizing the patient’s adjustment and development throughout follow-ups. SCNs can access the complete list of patients’ responses to each item immediately after completion, but these are not available to the patients.

### Study design

An exploratory inductive qualitative design was used. This paper is part of a larger study exploring patients’ experiences with a CFS in ostomy care. In this article, we address the first research question (RQ), focusing on their experiences with routine use of the CFS prior to consultations; a second RQ on patients’ experiences with the use of the CFS during the consultations themselves is addressed in a forthcoming article.

### Patients and recruitment

Patients were recruited from four outpatient clinics. Out of 30 invited patients, 27 responded. The selection ensured diversity in sex, age, type of ostomy, number of consultations, and scores. The average age was 59 years (range 23–83), with 13 males and 14 females. The group included 5 patients with a urostomy, 7 with an ileostomy, 13 with a colostomy, and 2 with multiple types of ostomies (Table 1). Most had curative treatments, while a few were receiving palliative care. Interviews occurred at varying intervals from the previous consultation.

**Table 1:** Informants demographics

*Patient No*	*Sex*	*Age*	*OAS score*^a^	*Time since surgery in months*^a^	*Number of consultations using OAS*	*Ostomy type*	*Hospital*	*Diagnosis*
*P1*	f	40s	≤ 2.9	18	2	Colostomy	1	Bacterial
*P2*	f	40s	≤ 2.9	12 years	5	Urinary reservoir/Colostomy	1	Functional diarrhoea
*P3*	m	70s	≤ 2.9	10	3	Colostomy	1	Incontinence
*P4*	m	70s	≤ 2.9	6 years	6	Urostomy/Colostomy	1	Radiation damage
*P5*	f	80s	≤ 2.9	28	6	Colostomy	1	Ca. recti
*P6*	m	70s	≤ 2.9	14	2	Colostomy	2	Ca. recti
*P7*	m	60s	≤ 2.9	13	3	Colostomy	1	Ca. recti
*P8*	m	70s	≤ 2.9	13	3	Urostomy	2	Ca vesica
*P9*	f	70s	≤ 2.9	15	3	Colostomy	2	Ca. recti
*P10*	m	60s	≤ 2.9	10	2	Urostomy	4	Ca. vesica
*P11*	f	70s	≤ 2.9	9	2	Colostomy	4	Diverticulitis
*P12*	m	40s	≤ 2.9	15	3	Ileostomy	2	IBD
*P13*	f	30s	≥ 3.0	10 years	6	Ileostomy	1	Hirschsprung disease
*P14*	f	40s	≤ 2.9	19	2	Ileostomy	1	IBD
*P15*	m	50s	≥ 3.0	14	2	Ileostomy	3	Ca. recti
*P16*	m	70s	≤ 2.9	14	2	Urostomy	3	Ca. vesica
*P17*	m	50s	≤ 2.9	27	4	Urostomy	2	Ca. vesica
*P18*	f	80s	≤ 2.9	14	3	Colostomy	2	Ca. recti
*P19*	f	60s	≤ 2.9	17	2	Urostomy	4	Bladder exstrophy
*P20*	f	60s	≥ 3.0	3	3	Colostomy	3	Diverticulitis
*P21*	f	20s	≤ 2.9	34	5	Ileostomy	1	IBD
*P22*	f	30s	≤ 2.9	18	3	Ileostomy	1	IBD
*P23*	f	60s	≤ 2.9	11	2	Colostomy	4	Incontinence
*P24*	m	50s	≥ 3.0	13	2	Colostomy	3	Ca. recti
*P25*	f	60s	≥ 3.0	15	3	Colostomy	3	Diverticulitis
*P26*	m	60s	≤ 2.9	4	2	Colostomy	4	Ca. recti

^a^Mean score ranging from 1 to 6. There is variation in the dataset. Scores lower than 4.35 indicate good adjustment, 2.67 to 4.34 some challenges, and 2.66 to 1 high adjustment

^a^Some patients had received an ostomy before the use of CFS in stoma care from 2017

### Data collection

The first author conducted individual semi-structured interviews in a private room at a public institution or the patient’s home between September 2023 and February 2024. The interview guide, developed from theoretical considerations, prior work, expert discussions, and literature review, was refined through test interviews. It covers two domains for the larger study: digital questionnaires and follow-up consultations. Minor adjustments were made after two test interviews, followed by a third. Interviews averaged 72 min (range 51–107 min) and were audio-recorded. Three patients had supplementary telephone interviews.

### Data analysis

The data were analysed using Braun and Clarke’s reflexive approach to thematic analysis, a six-phase process involving familiarization with the dataset, systematic coding, and developing, reviewing, and refining themes before producing the final report [29]. This approach is theoretically flexible and incorporates researcher subjectivity as integral to the analysis, viewing coding as an organic and flexible process requiring detailed engagement with the data. Themes are generated through coding and represent the outputs of the analytic process. Reflexivity involves critical reflection on our research roles, practices, and processes [29]. The analysis was conducted by the first author in close collaboration with the last author, involving patient representatives and research group at various stages to seek new insights and validation. The entire dataset was analysed in relation to two RQs. This article discusses the first, while the second will be presented elsewhere. See the supplementary materials for a detailed analysis description.

### Research team

LAJ, a Ph.D. candidate, is a trained therapist and psychiatric nurse with experience in qualitative research. AMS is a Professor in Nursing Science with extensive experience in qualitative research. KLI, with a Ph.D. in stoma care nursing, works as a SCN, and JRA is Professor in Nursing Science. All authors have critically reflected on their preconceptions and interests, approaching the data with an open mind.

Three patient user representatives recruited by Førde Hospital Trust participated in trial interviews and reviewed the results, providing feedback before translation into English.

### Ethics

This study was approved by the regional ethics committee (ID 593949) and the local data protection officers (references 4183). Anonymized transcripts were stored on secure servers. Patients received written information about the study’s purpose, anonymity, confidentiality, data security, and their right to withdraw without consequences, and written consent forms were signed. The interviewer asked participants about their interview experiences, and all reported positive experiences. No distress was noted.

## Results

Through analysis, the overarching theme *CFS—a tool with potential and multiple mechanisms of action* was developed. For the patients in this study, the CFS required them to consciously engage in subjective judgements; they did not experience it as eliciting objective measures of their condition. Engaging with the CFS had personal utility value with many dimensions. Underlying the patients’ use of the CFS was a desire to get help managing problem areas to better adapt to life with an ostomy. Three themes were developed: (1) Grasping the purpose can be challenging, (2) Preparatory learning—triggering reflection and self-awareness, and (3) Means of communication and potential for being understood.

All themes were represented in all interviews, but with varying strength and significance for each patient. The utility, or lack thereof, of the CFS depended on the patients’ needs and preferences in their process of adjustment to life with an ostomy.

### Grasping the purpose can be challenging

Grasping the purpose of the feedback system could be challenging for some. The patients exhibited a strong willingness to answer and understood that the SCN would receive or have access to the information, which was intuitive for many. Not everyone recognized that the questionnaire aimed to enable them to prepare for the consultation. Some patients perceived the questionnaire as solely for research aimed at improving clinical practice.

“It is for research, I suppose, ... about this [touches the ostomy]. I have kind of thought that it should go on to research.” (P9)

These patients did not explicitly articulate the significance of the questionnaire for their follow-up, but multiple mechanisms of action nonetheless emerged, which held personal significance for them through using the feedback system. They recalled receiving information about the CFS and its utilization through various channels, both verbally from the SCN and in written form, including the provided link to the questionnaire. Some mentioned receiving information through multiple channels, while a few reported not receiving any. Several patients found it challenging to absorb everything after surgery because of their reduced general condition, the influx of new impressions, and the large amount of information. Some experienced cognitive difficulties during subsequent treatments.

“I don’t think I received much information. I was so full of medications that I don’t even remember what day it was. I don’t think I got anything. I didn’t get anything about what it was supposed to be used for.” (P24)

Several patients explicitly stated that verbal information from the SCN was essential in understanding the purpose of the feedback system and could recall this information without remembering other details. One patient, who initially did not grasp the purpose of the questionnaire, emphasized the importance of

“ensuring that you understand that it [the questionnaire] is intended to help you as best as possible. It is not as crucial to understand how it works with graphs and such; that will be understood eventually. I do not know... papers and such... it’s important to have a verbal explanation before receiving the form.” (P13)

This patient was also eager to inform other patients so they could benefit from early follow-up. Internalizing knowledge about the purpose and use of CFS can lead to increased commitment and greater personal benefit.

### Preparatory learning—triggering reflection and self-awareness

By the process of answering the feedback items, the patients felt both invited and empowered to raise and discuss a wide range of topics in subsequent consultations. They also opined that the CFS provided the same opportunity for the SCN. Most patients expressed a sense of readiness for the consultation after completing the questionnaire. This process of preparatory learning made them feel better prepared by enabling them to develop a vocabulary around various topics.

“If it were not for the questionnaire... I wouldn’t have known what the follow-up would entail. I would probably have been like a question mark. I would, almost said, felt unprepared. When the SCN started asking about different things, I wouldn’t have reflected or had words to answer what she asked about. By answering this [questionnaire], I gain a better understanding of various topics and issues. I become more informed about what it involves and how to talk and discuss things.” (P7)

Answering feedback items prior to consultations enhanced patients’ ability to discuss their areas of concern with the SCN. This process also triggered a desire to orient themselves and acquire knowledge. They could search for information on websites for ostomy equipment, user organizations, or public information sites. Several patients initially believed that consultations with the SCN would be limited to physical examination of the ostomy. By responding to the questionnaire, they gained an understanding that the consultations could encompass more aspects of living with an ostomy, enabling dialogue on a broader range of topics—a new understanding of the breadth of issues that could be addressed.

“I realized I could discuss things beyond what I had initially thought... it was like a wake-up call—oh yes! I could bring up more than just, yes, about more things than the ostomy itself.” (P17)

Many patients found that answering questionnaires prior to each consultation allowed for self-reflection, leading to increased self-awareness of various aspects of life with an ostomy, including physical, psychological, and social factors.

“I discovered new aspects of myself, like my lack of patience. Now, I must think things through more. I have been thinking a lot about quality of life, especially considering that my physical condition is deteriorating. It makes me consider the quality of life … The fact that I am becoming more physically dependent and needing more assistance with the ostomy and everything, and the patience that requires.” (P3)

For some, self-reflection extended beyond life with an ostomy. They found that using the CFS provided insight into their inherent qualities and how they handled various situations. For a few, it prompted deeper reflection on what mattered most to them. Some participants were unsure how to respond to questions about quality of life when other health issues or impairments were more impactful than the ostomy itself. Similarly, questions about sexuality posed challenges for some when physiological obstacles or partner-related factors were involved. Some participants wished for the opportunity to provide additional context through free text comments to better explain their responses to the SCN. However, they considered that subsequent consultations could provide an opportunity to delve deeper into their answers.

### Means of communication and potential for being understood

By answering the questionnaire, the patients experienced the use as a means to communicate with the SCN. They experienced, or recognized the potential for, the CFS to provide the SCN with insight into their condition, feelings, and adaptation to life with an ostomy. This served as the foundation for further dialogue and provided opportunities for being understood.

“I respond exactly as I feel. It’s important for me to convey the situation accurately to the SCN… without glossing over anything. Using the questionnaire allows me to express myself effectively. Building upon this helps the SCN grasp the reality of my experiences.” (P2)

Using the feedback system made patients feel that the SCN knew them better. The opportunity to respond was perceived by several as an act of care by the SCN, indicating genuine concern for their well-being and contributing to the patients’ willingness to respond. For many, the CFS provided reassurance that essential topics could be addressed during consultations. Some saw it as a means to provide updates on their condition and reassurance that the SCN was monitoring their progress. Several patients found it easier to raise issues through the feedback system that they might otherwise have been reluctant to discuss verbally, particularly regarding sexuality and mental health. One patient with speech difficulties described it as “lifesaving” to communicate through the CFS.

“I’m asked about things I would not have dared to bring up otherwise. It is invaluable. It allows me to be completely honest about life with an ostomy.” (P13)

Patients generally found the questions understandable and easy to answer. Some noted the importance of paying attention to the wording of statements to respond accurately on the Likert scale. Assessing one's own knowledge of ostomy care and hernia risk could be difficult, and therefore also challenging to answer questions about. They found the questions relevant to their conditions and concerns, and the format consistent with acceptable time use.

“It is clear that the creators understood our struggles or experiences. The form is well-designed and thoughtful.” (P5)

Patients desired that the CFS provide an accurate depiction of their status so they could receive appropriate assistance in managing their issues and adjustment process. For some, it was a way to provide updates on their condition. Overall, they felt they could present themselves effectively using the CFS. Some, mostly men, found it challenging to provide a complete picture of sexuality. This was not only due to the complexity of the topic but also because their need for support might become significant after living with an ostomy for some time, which could be challenging to convey through the feedback system. One patient experienced underreporting on questions about leakage and noted discrepancies in his understanding of leakage after attending a coping course.

“The terminology matters. When I talked about leakage, I meant accidents, but when the instructor spoke, they referred to minor seepage under the bandage. So, I should have answered that it was more frequent, not just 1-2-3 times a month, but every day.” (P15)

Although patients felt they could honestly represent their situation through the feedback system, they acknowledged that simply reviewing the report by the SCN would not be sufficient to interpret all responses accurately. The complexity of their situations necessitated further exploration, elaboration, and discussion. Patients were motivated to engage in these discussions, covering all aspects, including sensitive topics. Some expressed reservations about the validity of the questionnaire if interpreted in isolation, without discussion between them and the SCN. Answering the feedback items was a subjective activity that required assessment and interpretation to represent themselves accurately.

## Discussion

This is the first study on how patients with an ostomy experience using a CFS in routine care. Despite difficulties in understanding its purpose, participants found the CFS to be a versatile tool with benefits such as preparatory learning, reflection, self-awareness, and communication.

### Promising, but grasping the purpose can be challenging

Even though patients received both written information and verbal instructions at the 3-week follow-up, the purpose can still be difficult to understand for some. They could recall receiving information, but not the content. Similar findings have previously been shown in rheumatology outpatient care, where patients did not remember receiving any information, and lack of knowledge affected their motivation to respond [30], a known user barrier [13]. Nevertheless, we found willingness, motivation, and capacity to respond. Even though the contexts are different, contributing factors for motivation in this study are relevant and likely include the high relevance of the questionnaire and its user-friendliness, both crucial factors for implementation [18, 31, 32].

A lack of knowledge or not being fully aware seems to be challenging for patients when using CFS across clinical settings [30, 33–35], and it may have consequences for how engaged individuals are, which in turn may affect the extent of the benefits of using the CFS. Gaps in understanding are naturally found to impede engagement. Therefore, health care workers must educate patients about the tool, but the literature provides little guidance on this [13]. Although some patients in this study still described a range of personal benefits to varying extents without grasping the purpose, we believe that the CFS can offer greater potential in supporting the adjustment process for some.

Lack of understanding can be explained from both the sender’s and the receiver’s perspectives, more likely in combination. We know from the literature that illness can make it difficult to comprehend, and that general health literacy can be affected [36]. The patients in this study mastered the CFS well, but there is a concern that digital questionnaires may contribute to disparities in healthcare [37, 38]. Healthcare professionals also require competence in health education to effectively communicate the purpose and, more generally, to improve patients’ health literacy, which encompasses various levels of comprehension and skills [39, 40]. This will be crucial, particularly with the expanding utilization of digital services and sustainability concerns for future healthcare delivery [39]. Unfortunately, many organizations lack practices that promote health literacy [39]. This CFS does not specifically measure patients’ health literacy [24], necessitating SCNs to assess this during clinical interactions. Tailoring information about the CFS’s purpose to align with patients’ health literacy levels places significant demands on SCNs as educators.

A significant finding was that engaging with the questionnaire provided preparatory learning with enhanced reflection and self-awareness. Nuances in how the CFS promoted reflection and awareness are also found in other studies [13, 41]. This study differs from others in how the CFS provides the patients with an understanding that the consultation could address a wider range of issues and aspects related to having a stoma. Through preparatory learning by engaging with the CFS, they felt prepared, empowered, and had an enhanced ability to discuss their areas of concern, encompassing physical, psychological, and social factors, with the SCN. Patients can obtain effective support for adaptation only if they are able to articulate their needs[4]. Patients gaining both understanding and self-competence can provide opportunities in consultations. Patient empowerment, defined as “the patients’ subjective sense of control over their own disease and treatment management,” is a concept deeply rooted in psychology [42]. Empowerment also requires healthcare services to share power, recognizing patients’ autonomy and responsibility [43].

Finally, patients viewed the CFS as a means of communication, enabling them to articulate their health status and serving as a foundation that provides the potential for being understood. This is important knowledge for SCNs on how to relate to the results, and that interpretation and exploration will be necessary for clinical utility. Numerous disparities exist in patient access to care [37]. If the CFS ensures communication, it might be a promising health technology to reduce disparities by overcoming barriers such as difficulty communicating with providers and inadequate communication between patients and providers regarding symptoms and health situations. However, it might potentially even widen disparities by yielding positive outcomes for everyone, but the strongest of those positive outcomes would be among patients who have enough education and cultural capital to take full advantage of the opportunities offered through the instrument.

Despite variations in strength and significance for each individual, responding to and engaging with the CFS does not appear to impose a burden [14]. This raises the question of whether it is ethically justifiable not to offer patients in ostomy care this opportunity, given the many benefits that appear in this study and the seemingly deliberate use of the CFS by SCNs in follow-up care.

### Limitations

The rich and varied data illuminate multiple aspects of the research question. To enhance reliability, we meticulously outlined the process of drawing conclusions, allowing readers to trace the analytical pathway. However, retrospectively exploring experiences can be challenging as it may not capture immediate experiences accurately. In line with the nature of qualitative research, the results cannot be generalized to all users of the CFS and must be understood in context. Furthermore, patients were recruited with instructions to ensure variation, not randomly, which introduces uncertainty regarding who was chosen and why. This limitation does not undermine the significance of our results, which we believe inform and expand upon the existing literature.

### Relevance to clinical practice and further research

The study provides valuable insights for SCNs in their practices, the further development of the CFS, and the development and use of CFSs in healthcare in general. Addressing patients’ educational needs in understanding the purpose and value of CFSs is crucial for clinical utility, and greater emphasis should be placed on this. Future research should identify the best ways to communicate and educate patients about using PROMs, determine which patient groups benefit most, and explore how patients with low health literacy respond to their use.

## Conclusions

Engaging with the CFS had personal utility value with many dimensions, varying in strength and significance for each patient. Even though not everyone grasped the purpose of its use, it was part of a preparatory learning process for consultations and the adjustment process itself. It triggered reflection and self-awareness and served as a means of communication with potential for follow-up. The study shows the need for and importance of communicating and educating patients about the purpose, value, and use of CFSs.

## Supplementary Information

Below is the link to the electronic supplementary material.

Supplementary file1 (DOCX 53 KB)

Supplementary file1 (DOCX 88 KB)

Supplementary file1 (PDF 419 KB)

## Data Availability

The data that support the findings of this study are not openly available due to reasons of sensitivity and are available from the corresponding author upon reasonable request.
