# Supplementary material for: Patients’ experiences with the routine use of a clinical feedback system prior to consultations in ostomy care: a qualitative study
Source: Qual Life Res. 2025 Feb 15;34(5):1473–82. doi: 10.1007/s11136-025-03916-z (PMC12064464; doi:10.1007/s11136-025-03916-z)
Supplement: Supplementary file 1 — Supplementary file1 (DOCX 53 KB) [file 11136_2025_3916_MOESM1_ESM.docx]

# Supplemental material – Analyses process

## Reflexive thematic analysis

The analysis was conducted by LAJ in close collaboration with AMS and involved all authors and patients service users at various stages to seek new insight and validation.

Phase 1: Familiarizing ourselves with the dataset

LAJ, who had conducted the interviews, transcribed all of them, becoming intimately familiar with and immersed in their content by repeated readings of the transcripts and listening to the audio file recordings. In this part of the analysis, LAJ engaged analytically with the data by assessing its potential to address the research question (RQ); What are the patients experiences with the use of a Clinical feedback system (CLF) as part of routine follow-up in ostomy care? Significant contents were marked in the audio files and on the margins of the transcripts, with brief notes made on ideas relating to individual data items and the entire dataset.

Phase 2: Coding

LAJ worked systematically through the entire dataset, identifying segments that were relevant to the RQ and applying analytically meaningful code labels. These labels encompassed both semantic and latent codes, capturing the researcher’s analytical perspective on the data. Segments from the text were cut out and affixed to file cards, annotated with a code name and informants’ numbers.

Phases 3: Generating initial themes

Initial themes were generated by identifying shared patterns of meaning across the dataset. This active process, led by LAJ in collaboration with AMS, involved clustering codes together based on their similarities to establish initial themes. A large wall was used to map out potential clusters. The relationships between the developing initial themes were reviewed, and coded data were systematically reviewed against the dataset to assist in potential theme identification. At this stage, five initial themes were identified.

Phase 4: Developing and reviewing themes

In this phase, the quality of the themes and boundaries between them were assessed. It became apparent that the themes were overly diverse and wide-ranging, lacking the necessary focus. They failed to capture the narrative in relation to the entire dataset and the RQ. The initial RQ was thus divided into two: RQ1, presented in this article, focuses on how patients experienced the routine use of the CFS prior to consultations; RQ2, on how patients experienced the use of CFS during consultations, is addressed in a forthcoming article. Consequently, LAJ undertook additional coding (phase 2) and revisited phase 3 to regenerate initial themes relevant to the revised RQs, resulting in two overarching themes, each linked to four and five initial themes, respectively. Upon returning to this phase, we ensured the themes aligned with the extracted codes and the full dataset before finalizing the thematic map.

Phase 5: Refining, defining and naming themes

By writing short abstracts of the themes, we ensured that the themes became clearly demarcated, constructed around robust core concepts, and integrated into the overall narrative about the data. We opted to revisit four initial themes for further review, which involved moving back and forth between the phases. This process led to the merging of two themes for both overarching themes to establish a core concept. As a result of this phase, we identified three themes addressing RQ1 and four themes addressing RQ2. Informative names for each theme were selected.

Phase 6: Writing up

Guided by abstracts from each previous phase, we composed our analytic narrative using relevant empirical extracts from the entire dataset. This was an integral part of the analytic process, involving extensive writing and rewriting in collaboration with the research group and service users.

**Fig** Overarching themes with initial codes from Phase 4. The initial codes for RQ2 are not displayed because they will be covered in a forthcoming article.
