# Supplementary material for: Patients’ experiences with the routine use of a clinical feedback system prior to consultations in ostomy care: a qualitative study
Source: Qual Life Res. 2025 Feb 15;34(5):1473–82. doi: 10.1007/s11136-025-03916-z (PMC12064464; doi:10.1007/s11136-025-03916-z)
Supplement: Supplementary file 2 — Supplementary file1 (DOCX 88 KB) [file 11136_2025_3916_MOESM2_ESM.docx]

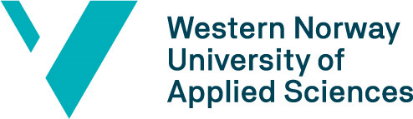

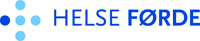


## Interview Guide: “Patient experiences with the use of a clinical feedback system in ostomy care”

- Thank you for participating.
- Information about rights and the interviewer
- Demographic data

*In the interview, I will ask questions about the follow-up you receive at the stoma and continence outpatient clinic (stoma care nurses) and living with a stoma. Gaining knowledge about how patients experience this is important to improve practice. You can speak freely about both positive and negative experiences. If I find something particularly interesting or relevant, I may ask additional questions or request further elaboration.*

## Adjustment and management

*Living with a stoma can, in many ways, be described as managing and adjusting to a chronic condition. How one does this is, of course, individual, and only you can say what it means for you.*

- Can you describe how you manage living with a stoma and how the process has been so far?
- What has been important for you up to now?
- What have you found helpful or less helpful?
- In the future, will anything be different for you?

## Digital QuestionnairEs

*Before you come for follow-up/consultation at the outpatient clinic, you receive a link on your phone or email with questions to answer.*

- How were you informed about the use of the questionnaire, and what information did you receive?
- What are your thoughts on the use of the questionnaire and its purpose?
- Can you describe how you experienced the questions and response options, and how it was for you to complete the questionnaire?
- How do the questions and topics correspond with your own experiences of problem areas?
- How was it to find a score that fit?
- Was there anything that prevented you from answering as you feel and experience?
- In your situation, what meaning does it have for you to answer the questions?
- What reflections did you make about your own situation by answering the questionnaire?
- Did you learn anything or become aware of something about yourself and your situation by answering the questions?
- Is there anything about the questionnaire you think should be different or changed?

## Follow-UP/CoNsULTATION

- Can you describe how the follow-up/consultation with the SCN [stoma clinical nurse] proceeds?
- To what extent and in what way did you feel the SCN was prepared?
- Did the SCN address topics that were important to you?
- Can you describe how the SCN used your responses during the follow-up/consultation?
- What did you find useful in the follow-up/consultation with the SCN? Anything not useful?
- Is there anything you wish were different with the follow-up?

## Communication – Relationship – interaction

- In what way does the use of the questionnaire affect how you and the SCN talk to each other?
- How do you assess the communication between you and the SCN?
- Does the questionnaire affect the relationship between you and the SCN – how?
- Does the questionnaire and the follow-up/consultation influence how you think about your own situation? In what way?

*Interaction between patients and healthcare professionals is often characterized by underreporting of symptoms and challenges of living with a stoma. Fear of leakage and difficulties with sexuality are especially underreported. This can be the case for both the patient and the stoma nurse. The questionnaire was introduced to improve communication so that patients can express difficult things and discuss them.*

- What are your experiences?
- Were any of the questions sensitive and difficult to answer for that reason?
- How does the questionnaire affect your ability to talk about what concerns you?
- Were any of your answers "red flags" or had "poor" scores – things that are challenging for you? How was this discussed during the consultation?
- Was there room to talk about other topics not covered in your answers?
- What prevents you from bringing up topics with the SCN?
- What would help you to be able to talk about difficult and personal topics?

## PErson-centred care – patienT involVement – quality

*Person-centred care is a goal – that you receive follow-up tailored specifically to you.*

- How do you feel the follow-up you receive is tailored to you and your needs?
- What role do you think the use of the questionnaire plays in being able to tailor the follow-up you receive?
- Were you offered or referred to other healthcare professionals because of your answers?
- How did you feel prepared for the consultation?
- How is it for you to prepare through such a questionnaire?

*Another intention of the questionnaire is for patients to participate more in their own treatment – that you make decisions together with the SCN, thereby increasing collaboration in the follow-up.*

- What are your experiences?
- How do you feel the follow-up and use of the questionnaire have affected how you think about your own situation and living with a stoma?

*Improving consultations is also one of the intentions of introducing the questionnaire.*

- What is your experience of the quality of the follow-up with the stoma nurse using the questionnaire?
- What do you think the difference would be between receiving follow-up with and without the use of this questionnaire?
- Thinking about what is important to you – how do you think the consultations and quality can be improved?

## SumMary

- In your view, what changes or adjustments would be wise to make to potentially increase the usefulness of using such a questionnaire as part of the follow-up?
- How willing are you to answer the questionnaire at future consultations?
- Is there anything we haven't discussed or anything you haven't had the chance to share?
- Do you have any questions?

## Questions after the Interview (debrief)

- How did you experience our meeting today?
- May I contact you again if I need additional information later?
